# Neuromuscular-related interventions for post-stroke dysphagia: a comprehensive narrative review

**DOI:** 10.3389/fnins.2026.1824363

**Published:** 2026-06-24

**Authors:** Muhan Zhou, Baoqiang Dong, Hongfei Zhou, Wei Zhang, Aihui Fu, Zhiqiang Sun, Xi Wu

**Affiliations:** 1 Liaoning University of Traditional Chinese Medicine, Shenyang, China; 2 Affiliated Hospital of Liaoning University of Traditional Chinese Medicine, Shenyang, China

**Keywords:** muscle training, neuromuscular electrical stimulation, neurostimulation, post-stroke dysphagia, swallowing rehabilitation

## Abstract

**Objective:**

To systematically review the efficacy, mechanisms, and application characteristics of neuromuscular-related interventions for post-stroke dysphagia (PSD), and to examine strategy selection across different swallowing stages to inform individualized rehabilitation strategies.

**Methods:**

PubMed, Web of Science, Embase, and MEDLINE were systematically searched for studies published between 1995 and 2025. Titles and abstracts were screened, and the full texts of eligible studies were retrieved for further analysis. Interventions related to neural and muscular regulation were categorized and synthesized into four main groups: exercise training and behavioral interventions; peripheral neuromuscular stimulation; central nervous system modulation techniques; and other adjunctive interventions.

**Results:**

A total of 293 publications were included, of which 56.63% were randomized controlled trials. The interventions were summarized into four major categories comprising more than 10 techniques. Exercise training and behavioral interventions (e.g., oral motor exercises) enhanced swallowing muscle strength and coordination. Peripheral neuromuscular stimulation (e.g., neuromuscular electrical stimulation and acupuncture) enhanced or modulated swallowing function by directly stimulating relevant nerves or muscles. Central nervous system modulation techniques (e.g., transcranial magnetic stimulation and transcranial direct current stimulation) influenced swallowing-related neural networks indirectly by regulating cortical excitability. Other adjunctive interventions included botulinum toxin injection, which directly targeted the cricopharyngeal muscle. Further analysis examined the selection of key rehabilitation techniques across different clinical stages of PSD, integrating central and peripheral neuromodulation approaches. It explored the potential implications of soft-tissue surgery and meridian-muscle theory for PSD management to inform individualized clinical decision-making.

**Conclusion:**

Neuromuscular interventions were found to be widely used in PSD management, particularly transcranial magnetic stimulation, acupuncture, and neuromuscular electrical stimulation. Future strategies should integrate pathology, clinical manifestations, and lesion localization to develop central lesion–oriented multimodal therapies that combine peripheral nerve and muscle interventions, potentially improving clinical outcomes.

## Introduction

Post-stroke dysphagia (PSD) refers to impaired swallowing resulting from damage to central nervous system structures that disrupts the normal function of swallowing muscles, preventing food from being safely and efficiently transported to the stomach. PSD may lead to malnutrition, dehydration, aspiration pneumonia, and depression (Warnecke et al., 2021). Among these complications, aspiration pneumonia is a major contributor to increased mortality after stroke. Approximately 12.2 million stroke events occur worldwide each year (Prust et al., 2024), and nearly 50% of patients develop dysphagia (Ehsaan et al., 2023). PSD is therefore one of the most common complications of stroke (Umay et al., 2022) and an independent predictor of poor prognosis.

PSD treatment includes dietary interventions, behavioral therapy, pharmacotherapy, and neural stimulation (Dziewas et al., 2021). Simple adjustments to food consistency often result in inadequate nutritional intake (Murray et al., 2016). Pharmacological management involves antibiotics and prokinetic agents; however, antimicrobial therapy lacks large-scale supporting data and has limited efficacy in preventing aspiration pneumonia, while prokinetic drugs such as metoclopramide remain underutilized clinically (Burgos et al., 2018). Consequently, current treatment strategies primarily rely on behavioral interventions and neural stimulation. Behavioral interventions are often combined with task-oriented exercise training to enhance the function of specific swallowing muscles. Neural stimulation can be divided into peripheral and central approaches: peripheral stimulation directly targets swallowing muscles and their associated peripheral nerves, whereas central stimulation modulates swallowing-related neural networks. Additionally, botulinum toxin may be employed to target neural structures and reduce cricopharyngeal muscle tone.

The swallowing process consists of oral, pharyngeal, and esophageal phases (Park et al., 2016), involving the coordination of 26 pairs of facial muscles and at least five cranial nerves (Moore et al., 2014), thereby making soft-tissue coordination critical. Patients with PSD may improve swallowing function by adjusting soft tissue structure and tension. Soft tissue surgical techniques had been widely applied to treat dysfunctions such as spasticity following central nervous system injury; however, systematic reviews integrating these principles into neuromuscular interventions for PSD were still lacking.

Several secondary research syntheses provided evidence-based foundations for PSD interventions. Geeganage et al. (2012) conducted repeated systematic reviews on dysphagia in acute and subacute stroke, while Speyer et al. (2010) and Pisegna et al. (2016), and the ESO-ESSD guideline (Dziewas et al., 2021) respectively offered evidence-based support for behavioral interventions, non-invasive brain stimulation, and comprehensive management of PSD. However, these works each focused on specific intervention categories. A comprehensive review that spans peripheral neuromuscular stimulation, central neuromodulation, and exercise-based training, and that integrates these techniques within a unified analytical framework, has remained lacking. Building on this, the present study reviews the first systematic review of PSD neuromuscular stimulation techniques, summarizing the efficacy, mechanisms of action, and application characteristics of various interventions. The discussion examines treatment selection and combined therapy strategies for swallowing dysfunction across different phases, and suggests potential future directions for personalized precision rehabilitation.

## Data sources and methods

### Research question

This study formulated the following question to guide the scoping review: What are the efficacy and mechanisms of neuromuscular interventions for PSD?

### Literature search

All information sources included PubMed, Web of Science, Embase, and MEDLINE via EBSCOhost. The search strategies were adapted for the indexing structure and syntax of each database platform and are presented in full in the Appendix. The core concepts combined were stroke, dysphagia, and the neuromuscular interventions listed below. Two authors independently conducted the searches. The initial literature search was conducted on 20 October 2025. No restrictions were applied regarding language or publication year. The following terms were used as subject headings, keywords, and MeSH terms to construct the search strategy:

The search terms included “Stroke,” “Deglutition Disorders,” and various swallowing rehabilitation interventions, “Oral exercise,” “Electromyography Biofeedback,” “Transcutaneous Electric Nerve Stimulation”, “Acupuncture”, “Transcranial direct current stimulation”, “Transcranial Magnetic Stimulation”, etc. The full search strategies for each database are provided in the Appendix.

### Literature screening

Two authors independently screened titles and abstracts according to predefined inclusion criteria. Studies involving patients with ischemic or hemorrhagic stroke and a concurrent diagnosis of PSD were considered eligible. Studies were manually excluded if they did not meet diagnostic criteria, were unrelated to clinical PSD treatment, did not involve neuromuscular interventions, or were non-therapeutic studies. Screening criteria were finalized through consensus among all authors. For eligible studies, two reviewers independently extracted and analyzed data. A third author resolved any discrepancies through manual reference checks and review of the intervention studies.

### Classification of interventions

This review categorized interventions according to their therapeutic mechanisms. Exercise training and behavioral interventions included oral motor training, chin tuck against resistance, Mendelsohn maneuver, Shaker exercises, expiratory muscle training, biofeedback, and catheter balloon dilation. Peripheral nerve stimulation interventions included neuromuscular electrical stimulation, pharyngeal electrical stimulation, transcutaneous auricular vagus nerve stimulation, acupuncture, and peripheral sensory stimulation. Central nervous system stimulation interventions included repetitive transcranial magnetic stimulation and transcranial direct current stimulation. Adjunctive or alternative interventions included botulinum toxin therapy.

### Literature information extraction

**Figure 1:**
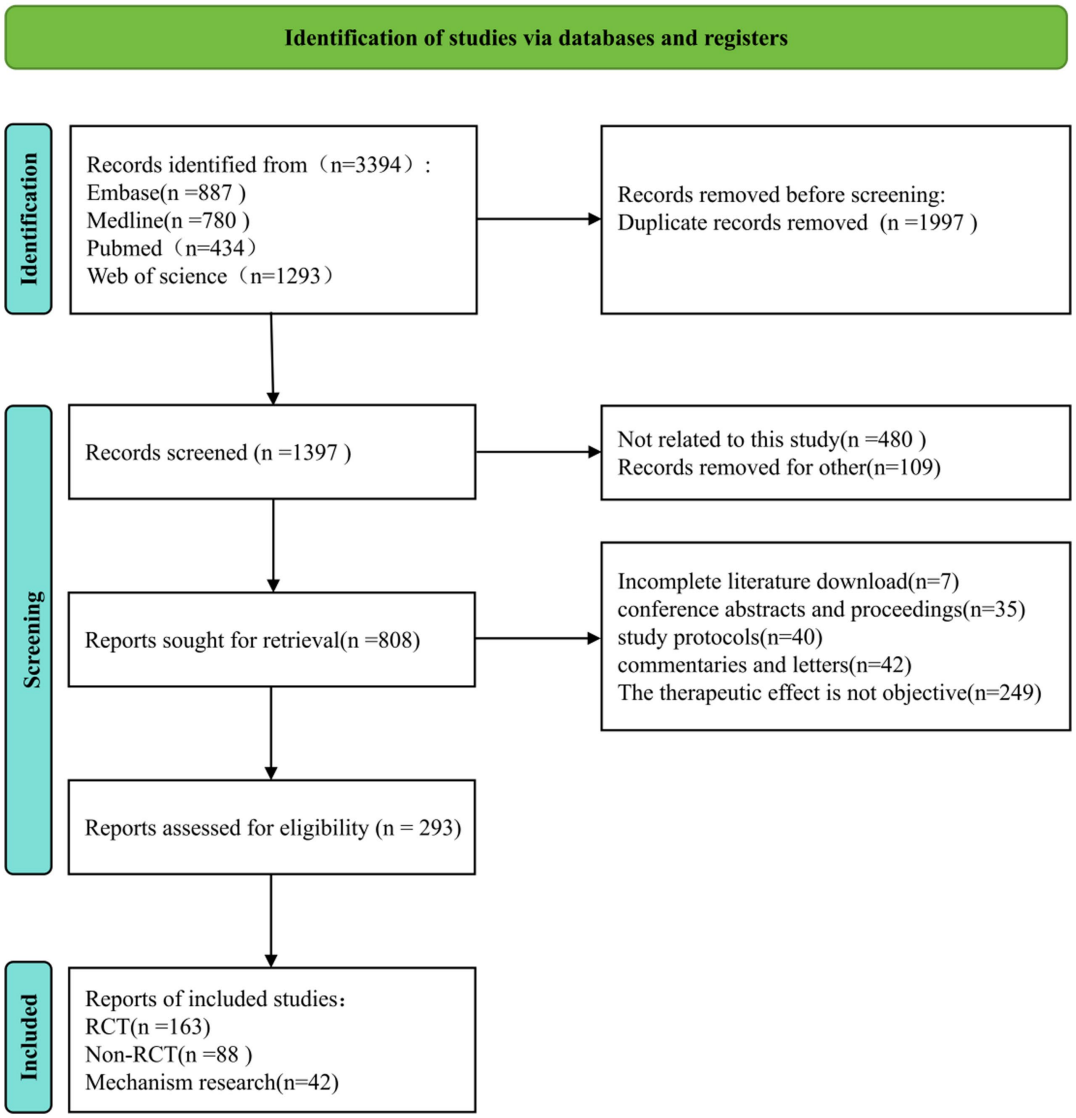
Identification of studies via databases and registers.

Two authors independently read all articles included in this review. Following this, data were extracted into Microsoft Excel according to predefined requirements. Extracted information included the article title, first author, study type, publication year, and a summary of content. A third author subsequently verified all data. The study screening process and results are presented in Figure 1 and Supplementary Table S1.

## Results

### Study characteristics

**Figure 2:**
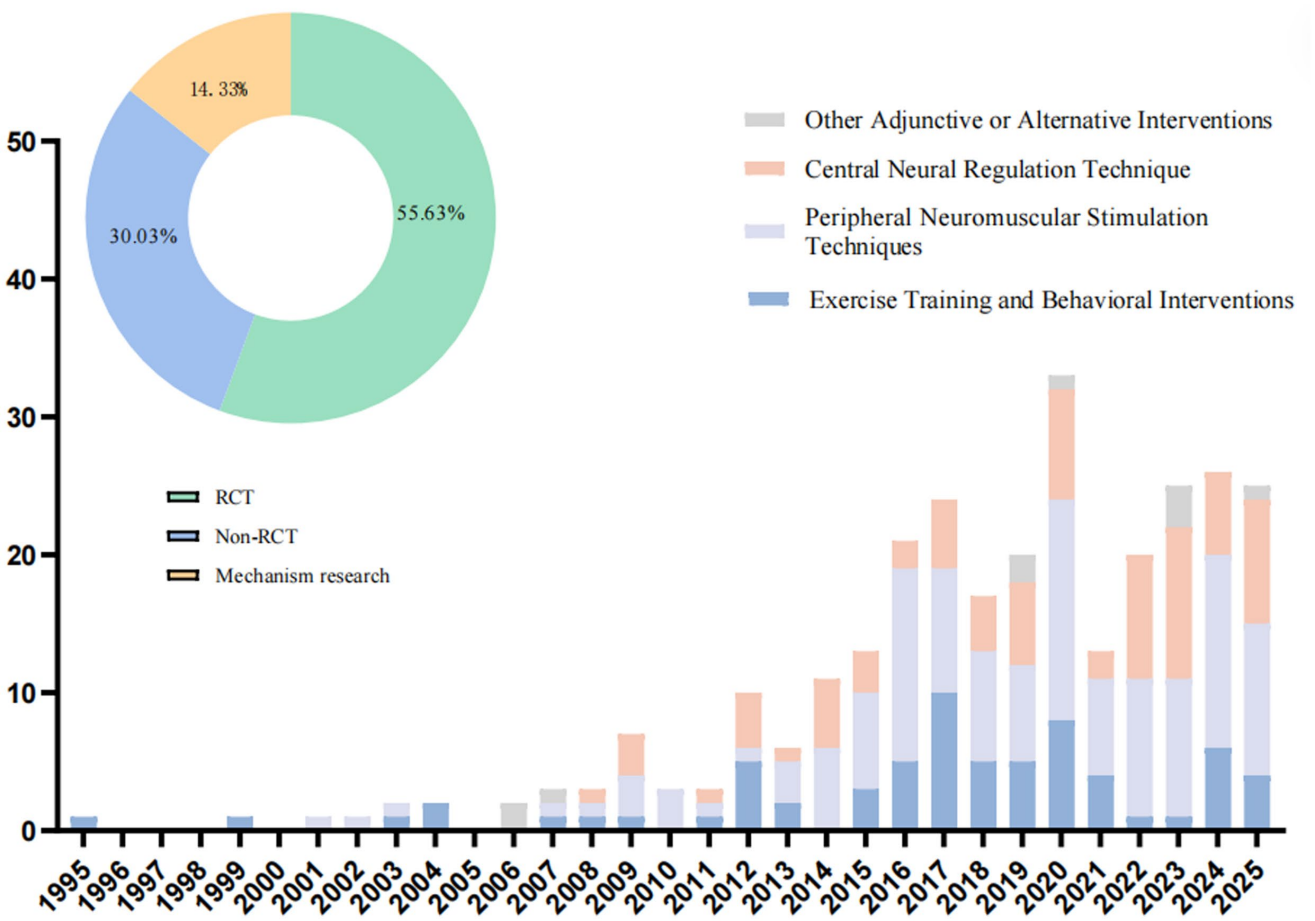
Publication frequency statistics.

After deduplication, the initial screening yielded 1,397 articles. Based on title and abstract review, 589 articles were excluded for not meeting the inclusion criteria. Following full-text review, an additional 515 articles were excluded, leaving 293 articles included in the final synthesis. Reasons for exclusion at the full-text stage included:

(1) Incomplete article download (inability to obtain full texts despite exhaustive attempts, e.g., interlibrary loan requests, contacting corresponding authors via email, and searching ResearchGate), with a total of 7 studies excluded for this reason. We acknowledge that no language, country, or journal restrictions were applied in our search strategy, and the exclusion of these unavailable articles is unlikely to introduce systematic bias due to their small number and diverse origins; (2) conference abstracts and proceedings; (3) study protocols; (4) commentaries and letters; (5) studies without validated objective outcome measures for swallowing function (e.g., relying solely on patient self-report without instrumental or clinician-rated assessments), or those in which the intervention did not directly target PSD; (6) secondary research (e.g., literature reviews, systematic reviews, meta-analyses). The 293 included articles were categorized by study type: randomized controlled trials (RCTs) (55.63%), non-RCTs (30.03%), and mechanism studies (14.33%) (Figure 2). Non-RCTs comprised observational studies, cohort studies, case series or reports, and pre-post self-controlled studies. Mechanism studies included animal experiments and neurophysiological studies in healthy subjects (Parkkinen et al., 2018). Publication years ranged from 1995 to 2025, with 77% published within the last decade.

**Figure 3:**
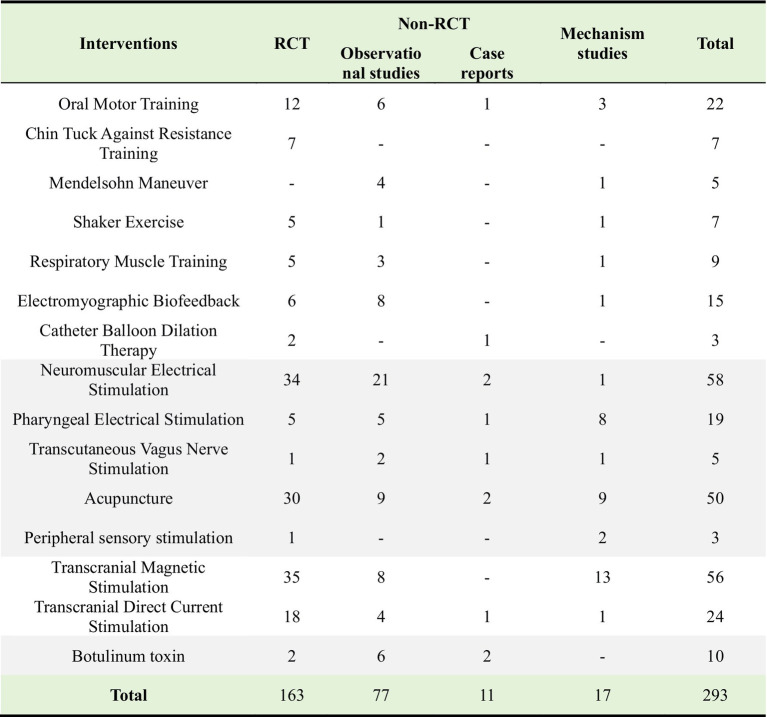
Statistical table of intervention studies.

When classified by intervention type, the included studies comprised: exercise training and behavioral interventions (23.21%), peripheral neuromuscular stimulation techniques (46.08%), central nervous system modulation techniques (27.30%), and other adjunctive or alternative interventions (3.41%). Analysis of the included studies revealed that most publications focused on neuromuscular electrical stimulation (NMES) and transcranial magnetic stimulation (TMS). From the perspective of research geography, China contributed the largest number of studies, with 122 publications accounting for 41.6% of the included literature, followed by South Korea (55 studies, 18.8%), the United States (25 studies, 8.5%), the United Kingdom (19 studies, 6.5%), and Japan (7 studies, 2.4%). These findings indicate that East Asia, North America, and Europe are the major regions contributing evidence in this field. Chinese studies were mainly concentrated on acupuncture and transcutaneous auricular vagus nerve stimulation, whereas studies from South Korea focused more on exercise training and behavioral interventions. In contrast, research from Europe and North America more frequently addressed neuromuscular electrical stimulation, transcranial magnetic stimulation, pharyngeal electrical stimulation, and device-based interventions (Figure 3).

### Exercise training and behavioral interventions

This section provides a detailed overview of seven intervention techniques aimed at improving PSD by strengthening and coordinating the swallowing muscles.

#### Oral motor training

Oral Motor Training (OMT) is an intervention used to improve PSD by targeting and strengthening swallowing-related muscle groups. It involved modulation of muscle tone, strength, range of motion, and coordination among multiple muscles, including the tongue, orbicularis oris, masseter, and buccinator. Its core mechanism was associated with muscle dynamics and fascial force transmission (Hwang et al., 2019). Studies showed that tongue pressure below 40 kPa represented a threshold for aspiration risk (Lee and Choi, 2020). Progressive tongue resistance training significantly increased anterior and posterior tongue pressures and reduced epiglottic vallecular residue (Krekeler et al., 2023; Robbins et al., 2007). Resistance jaw opening exercise (RJOE) improved hyoid movement and swallowing safety, with particularly pronounced benefits during liquid swallowing (Park et al., 2020). Tongue pressure strength and accuracy training (TPSAT) enhanced the pressure gradient between the tongue root and the posterior pharyngeal wall through isometric resistance, resulting in a 13–18% increase in maximum isometric tongue pressures (MIPs) after 8 weeks (Moon et al., 2018). Passive stretching of the tongue to maximal protrusion also improved tongue range of motion and oral-phase swallowing function (Pisegna et al., 2016).

In addition, pharyngeal strengthening combined with hyolaryngeal movement exercises enhanced pharyngeal and laryngeal muscle strength, thereby improving airway protection, reducing Penetration-Aspiration Scale (PAS) scores, and increasing Functional Oral Intake Scale (FOIS) scores (Kusumaningsih et al., 2019). Kinesiology taping (KT)-assisted effortful swallowing training could activate suprahyoid muscles, leading to improved pharyngeal-phase performance on videofluoroscopic swallowing study (VFSS) and reduced PAS scores after 6 weeks (Kim and Park, 2024). The hyoid bone is connected to the skull, mandible, and shoulder girdle, and indirectly to the pelvis, serving as a pivotal structure in swallowing. Following orofacial regulation therapy combined with body regulation, swallowing speed increased by 59%, and the duration of oral pressure maintenance was extended to 11.8 s (Hägg and Larsson, 2004). At the neuromodulatory level, tongue muscle reconstruction relied on crossed innervation from the infarcted hemisphere. Larger infarct areas were associated with more pronounced expansion of the contralateral mandibular motor representation area and greater atrophy of the tongue motor representation area in the infarct hemisphere (Cullins and Connor, 2024). Lip muscle training triggered a “lip-closure–tongue-retraction” coupling through the trigeminal (V)–facial (VII)–hypoglossal (XII) neural pathway, activating coordinated function of the buccinator and suprahyoid muscles (Hägg and Anniko, 2008). Voice training improved glottic closure by enhancing contraction of the lateral cricoarytenoid muscle (Fraga et al., 2018).

#### Chin tuck against resistance training

Chin tuck against resistance (CTAR) training strengthens the contractile force of the suprahyoid muscle group, thereby elevating the hyoid toward the larynx and facilitating epiglottic inversion to promote airway closure (Park et al., 2018). When combined with conventional rehabilitation approaches, CTAR could further enhance therapeutic outcomes (Lee et al., 2025). In a small randomized controlled trial (RCT), 22 patients with PSD were assigned to either a CTAR group or a conventional therapy group. After 4 weeks of intervention, the CTAR group demonstrated greater improvements in oral muscle strength, laryngeal elevation, and epiglottic closure, with less residue in the oropharynx and pyriform sinuses compared with the control group (Gao and Zhang, 2016). Another RCT comparing game-based CTAR with Shaker exercise reported higher completion rates and a lower psychological burden in the CTAR group (Park et al., 2019b). Multi-directional CTAR (md-CTAR), which incorporated diagonal and vertical movements in addition to sagittal-plane motion, showed superior improvements in tongue thickness and tongue pressure compared with conventional CTAR (Park et al., 2024). Kim and Park (2019) secured the CTAR device to a tabletop to minimize upper-limb compensation during training.

#### Mendelsohn maneuver

The Mendelsohn maneuver (MM) improved swallowing function by actively prolonging laryngeal elevation during swallowing, thereby enhancing movement of the hyoid–laryngeal complex and facilitating upper esophageal sphincter (UES) opening and pharyngeal contraction. A key advantage of this maneuver was sustained muscle activation, which prolonged UES opening time and facilitated bolus passage (Kim et al., 2017). McCullough et al. (2012) found that after 2 weeks of MM therapy, the duration of maximum hyoid elevation (DOHME) and the duration of maximum hyoid anterior excursion (DOHMAE) were significantly prolonged, with the therapeutic effects increasing over time. Subsequent studies further confirmed that MM increased the magnitude of hyoid elevation (MHE) and maximum hyoid anterior excursion (MHAE), prolonged UES opening duration, and improved the coordination of bolus transport (McCullough and Kim, 2013). Won (2012) combined oral sensory stimulation and manual facilitation technique (MFT) with MM in patients with severe PSD who were unable to initiate swallowing voluntarily. Palpation-based stimulation of the anterior belly of the digastric muscle and the thyrohyoid muscle, together with cold–acid oral sensory stimulation (4 °C lemon juice and frozen tongue-depressor stimulation at the tongue base), successfully elicited swallowing responses in three patients, suggesting that multimodal interventions could strengthen neuromuscular control and promote long-term recovery of swallowing function.

#### Shaker exercise

Shaker exercise (SE) required patients to lie in a supine position and lift the head to look toward the feet while keeping the shoulders off the bed. Through contraction of the thyrohyoid, mylohyoid, geniohyoid, and the anterior belly of the digastric muscles, the exercise induced anterior–superior displacement of the hyoid–laryngeal complex, thereby strengthening the suprahyoid and infrahyoid muscle groups and promoting opening of the UES (Kim et al., 2015). An RCT demonstrated that SE, combined with conventional therapy, significantly improved PAS and FOIS scores compared with conventional therapy alone, confirming that SE effectively reduces aspiration risk and improves oral intake by strengthening the anterior cervical musculature (Choi et al., 2017). Proprioceptive neuromuscular facilitation (PNF) shared core movement patterns with both CTAR and Shaker exercise, targeting the suprahyoid and infrahyoid muscles through multidirectional movements; however, unlike CTAR and SE, PNF activated these muscles primarily through stretching, resistance training, and proprioceptive stimulation. Despite these shared movement patterns, two separate RCTs demonstrated that PNF was superior to both CTAR and Shaker exercise in tongue base and hyoid elevation, epiglottic closure, pyriform sinus residue, post-swallow pharyngeal wall residue, pharyngeal transit time, and aspiration (Özcan et al., 2025; McCullough et al., 2012).

#### Respiratory muscle training

Respiratory muscle training (RMT) improved swallowing by strengthening respiratory muscles through targeted breathing exercises. It mainly included expiratory muscle strength training (EMST) and inspiratory–expiratory muscle training (IEMT). The underlying mechanism involved activation of the suprahyoid muscles, which facilitated hyoid elevation (Dziewas et al., 2021; Eom et al., 2017). An RCT demonstrated that EMST significantly enhanced suprahyoid muscle activity in patients with PSD, thereby reducing aspiration and improving oral intake (Park et al., 2016). Liaw et al. (2020) reported in an RCT that combined inspiratory muscle training (30–60% maximal inspiratory pressure, MIP) and expiratory muscle training (15–75% maximal expiratory pressure, MEP), used as an adjunct to conventional rehabilitation, significantly improved maximal inspiratory pressure, forced vital capacity, forced expiratory volume in 1 s, and fatigue. Acoustic analyses further showed concurrent improvements in shimmer, amplitude perturbation quotient (APQ), and voice turbulence index (VTI). Subsequent studies incorporating surface electromyography (sEMG) indicated that 6 weeks of RMT improved electrical activity in affected swallowing muscles, with the RMT group outperforming the conventional rehabilitation group during dry swallowing (Liaw et al., 2021). Additional research showed that combined RMT could increase peak expiratory flow by 168%, effectively reducing penetration–aspiration while simultaneously improving oral intake and swallowing function and strengthening airway protection (Arnold and Bausek, 2020). A single-blind RCT further demonstrated that the IEMT group showed significantly greater improvements in respiratory muscle strength compared with conventional therapy and neuromuscular electrical stimulation groups (Guillén-Solà et al., 2017).

#### Electromyographic biofeedback (EMGBF)

Electromyographic biofeedback (EMGBF) monitored the electrical activity of the submental and suprahyoid or infrahyoid muscle groups in real time. It converted these signals into audiovisual feedback, enabling patients to perceive and voluntarily regulate swallowing-related neuromuscular activity. It served as an important tool for assessing peripheral neuromuscular function and guiding motor training in PSD (Crary, 1995). The therapeutic effects of EMGBF were mainly attributed to two mechanisms. First, as a feedback modality, it compensated for impaired proprioception and refined movement patterns through operant conditioning (Huckabee and Cannito, 1999). In this context, electromyography was also used to quantify the effects of kinesiology tape (KT), demonstrating that KT significantly increased the average electromyographic amplitude (aEMG) of the suprahyoid muscle group (Wu et al., 2025). Second, as a therapeutic intervention, EMGBF delivered low-frequency electrical stimulation that promoted coordinated muscle contraction and increased oropharyngeal pressure (Meng et al., 2024). When combined with KT, stimulation of cutaneous receptors might further recruit muscle fibers and enhance neural activation, resulting in superior therapeutic efficacy compared with KT alone (Wang et al., 2020).

Surface electromyographic biofeedback (sEMGBF) was the most commonly used form of electromyographic biofeedback (EMGBF) as an adjunctive therapy for dysphagia. In clinical practice, sEMGBF combined with conventional rehabilitation strategies (Nordio et al., 2022), such as the effortful swallow, supraglottic swallow, the Masako maneuver, or the Mendelsohn maneuver (Alyanak et al., 2025), was shown to effectively reduce pharyngeal residue and lower the risk of aspiration. In addition, sEMGBF integrated with dual-module “strength and skill” training (BASSKIT system) was shown to improve Dysphagia Severity Rating Scale (DSRS) scores in acute PSD (Benfield et al., 2023) and to more accurately identify patients at high risk of persistent dysphagia based on underlying pathophysiological deficits. In patients with chronic PSD (Girod-Roux et al., 2025), adjunctive sEMGBF therapy improved FOIS scores, with reported tube removal rates of up to 54.5% (Bogaardt et al., 2009). Furthermore, sEMG also provided mechanistic insights into swallowing physiology, as studies showed that submental muscle electromyographic amplitudes during effortful swallowing were significantly higher than during natural swallowing (*p* < 0.001) in both PSD patients and healthy individuals, independent of age (Archer et al., 2021).

#### Catheter balloon dilatation therapy

Catheter balloon dilatation therapy primarily targeted cricopharyngeal muscle dysfunction by mechanically dilating the UES (Wei et al., 2017). An RCT demonstrated that conventional rehabilitation combined with pyriform sinus balloon dilatation for 4 weeks significantly reduced pharyngeal residue and pharyngeal transit time, and markedly improved PAS and VDS scores, without adverse events. This intervention might enhance UES relaxation by sensitizing the pharyngeal plexus, including the pharyngeal branches of the vagus and glossopharyngeal nerves (Kim et al., 2018). Bogaardt et al. (2009) further guided patients to perform autonomous swallowing using graded balloon dilatation; by leveraging the physiological opening of the UES during swallowing, the balloon was able to move within the sphincter, thereby not only effectively dilating the cricopharyngeal muscle but also synchronously stimulating the pharyngeal musculature. Consequently, the UES opening diameter (*p* < 0.001) and hyoid displacement (HD, *p* = 0.03) were significantly increased. Mechanistically, this approach might activate the corticobulbar pathway, as evidenced by a significant increase in motor-evoked potential (MEP) amplitudes of the submental muscles on the affected side (*p* = 0.02). Case reports suggested that balloon dilatation might be effective for refractory UES dysfunction following brainstem infarction, facilitating gastrostomy tube removal and restoration of oral feeding (Willert et al., 2003).

### Peripheral neuromuscular stimulation techniques

This section summarizes five intervention techniques that directly target the relevant nerves or muscles to enhance or modulate the function of the swallowing musculature.

#### Neuromuscular electrical stimulation (NMES)

Neuromuscular electrical stimulation (NMES) was a non-invasive therapeutic modality that facilitated the recovery of swallowing function by delivering electrical stimulation to weakened or paralyzed cervical muscles, thereby targeting the peripheral neuromuscular system (Byeon and Koh, 2016). Stimulation of the masseter and suprahyoid muscle groups improved oral-phase swallowing difficulties (Lee et al., 2019). Zhang et al. (2022) attempted to target the suprahyoid muscles and the C7 spinous process and reported that this approach shortened oral transit time and enhanced pharyngeal initiation to prevent aspiration. The suprahyoid muscles primarily elevated the larynx and hyoid bone, whereas the infrahyoid muscles were mainly responsible for their depression (Matos et al., 2022). Applying resistive stimulation to the infrahyoid muscles reduced aspiration risk and decreased pharyngeal residue (Oh et al., 2020; Beom et al., 2015). Single-point suprahyoid stimulation and dual-point stimulation spanning the suprahyoid and infrahyoid regions yielded comparable overall efficacy (Meng et al., 2018; Huh et al., 2020). However, conventional dual-channel synchronous stimulation could induce downward displacement of the laryngo-hyoid complex, producing movements opposite to those required for swallowing. Sequential 4-channel NMES, which triggered the suprahyoid muscles, thyrohyoid muscle, and infrahyoid muscles in physiological order, optimized laryngo-hyoid complex movement (Lim et al., 2023; Lee et al., 2021). Regarding stimulation parameters, typical settings included a frequency of 80 Hz and pulse widths of 300–700 μs, with shorter pulses possibly requiring higher intensities to activate target muscles (Grill Jr and Mortimer, 1996). Short pulses (300 μs) might transiently enhance maximal tongue pressure, but by the second week, their effect is comparable to longer pulses (700 μs; Takahashi et al., 2022). Stimulation intensity was gradually increased from 2.5 mA to the patient’s tolerance (usually ≤25 mA); excessive current might preferentially recruit type II fibers over physiologically normal type I fibers, resulting in reverse recruitment and reduced training efficiency (Carnaby et al., 2020). The most common treatment duration per session was 30 min; longer sessions might be counterproductive due to rapid fatigue of type II fibers (Huh et al., 2020).

NMES was a form of transcutaneous electrical stimulation that targeted muscle tissue. According to the stimulation parameters applied over the target muscle region, NMES could be further classified into sensory NMES and motor NMES. Sensory NMES was considered to act predominantly through modulation of central neuroplasticity (Gallas et al., 2010). Strengthening ascending sensory input drove plastic reorganization of the cortex, thereby contributing to the repair of the swallowing reflex arc and the establishment of new motor pathways (Oh et al., 2020). In contrast, motor NMES aimed to strengthen peripheral muscles and facilitate neural activation. By eliciting action potentials in motor neurons, it enhanced the contractile force of the suprahyoid and thyrohyoid muscles, thereby increasing the excursion of the hyolaryngeal complex (Zeng et al., 2018). In addition, it might prevent disuse atrophy of denervated swallowing muscles dominated by type II fibers (Wang et al., 2014) and promote neural regeneration, thereby facilitating reconstruction of the swallowing reflex arc. One RCT showed that sensory stimulation (low intensity, 0.25 Hz) enhanced oropharyngeal sensory input, facilitated reorganization of the central pattern generator and cortex, and achieved better outcomes than motor stimulation (120 Hz) and traditional dysphagia training (TDT) alone (Zhang et al., 2016). Another RCT found that sensory-level stimulation (≤3–4 mA) was associated with greater improvement in swallowing function scores than motor-level stimulation (maximum tolerated intensity), although the between-group difference did not reach statistical significance (Howard et al., 2023). Arreola et al. (2021) further suggested that sensory stimulation might enhance airway protection by increasing cortical excitability. However, other studies reported that motor NMES might be more effective in improving overall swallowing function, whereas sensory stimulation appeared superior and safer in reducing PAS scores, suggesting that motor stimulation might confer benefits unattainable with sensory stimulation alone through structural effects and stronger central activation (Rofes et al., 2013). Therefore, owing to heterogeneity in stimulation parameters and treatment duration in the current literature, the relative superiority of these two modes remained inconclusive. With respect to combined interventions, NMES combined with TDT was shown to significantly improve swallowing safety, shorten oropharyngeal transit time, and facilitate tube removal (Tarihci Cakmak et al., 2023; Simonelli et al., 2019; Xu et al., 2024), whereas no significant difference was observed when either treatment was used alone (Li et al., 2018). Mechanistically, NMES preferentially targeted type II fast-twitch muscle fibers, whereas TDT primarily recruited type I slow-twitch fibers (Zhang and Wu, 2021; Kushner et al., 2020); when combined, these modalities might generate greater contractile force in the swallowing musculature (Wang et al., 2023). In addition, NMES combined with resistance training (Sproson et al., 2018), thermal-tactile stimulation (TTS) (Lim et al., 2009), the Mendelsohn maneuver (Byeon, 2020), electromyographic biofeedback (EMG-BF) (Park et al., 2019a), oral facial rehabilitation (OFR) (Konecny and Elfmark, 2018), or transcranial direct current stimulation (tDCS) (Bengisu et al., 2024) demonstrated greater efficacy than monotherapy in improving swallowing function and quality of life.

#### Pharyngeal electrical stimulation (PES)

Pharyngeal electrical stimulation (PES) activated mucosal sensory nerves via a pharyngeal electrode catheter (Hu et al., 2024). The core mechanisms of PES involved the induction of substance P (SP) release (Suntrup-Krueger et al., 2016) and the reorganization of cortical excitability within the swallowing network (Jayasekeran et al., 2010). Concurrently, stimulation of afferent sensory fibers in the nasopharyngeal mucosa activated the glossopharyngeal and vagus nerves, thereby facilitating nucleus tractus solitarius–cortical plasticity (Bath et al., 2020). Essa et al. (2017) provided preliminary evidence that responsiveness to PES might be associated with the Val66Met polymorphism of the BDNF gene; with carriers of the Met allele showing greater functional improvement at 3 months. The authors proposed that neural remodeling induced by electrical stimulation may depend, at least in part, on BDNF-related pathways.

With respect to stimulation parameters, an optimal frequency of 5 Hz was identified (Hu et al., 2024; Harvey et al., 2025), which was closely associated with swallowing network plasticity. Conventional PES employed a pulse width of 0.2 ms, whereas modified PES (mPES) utilized a 10 ms hybrid waveform to enhance central sensory afferent input (Zhang et al., 2023; Zhang et al., 2023). Stimulation intensity was individualized according to patient tolerance, with a mean range of 14.8–28.28 mA; intensities below 15 mA were associated with neutral outcomes (Muhle et al., 2018; Bath et al., 2016). The optimal protocol consisted of 10 min per session for three consecutive days (Bath et al., 2020; Zhang et al., 2023), while prolonged treatment durations might attenuate therapeutic benefits (Vasant et al., 2016). Clinically, the primary benefit of PES lies in facilitating decannulation. Multiple RCTs demonstrated that the decannulation rate in the PES group was approximately 40% higher than that in sham-stimulation controls (Dziewas et al., 2018). In critically ill patients, PES achieved decannulation rates of up to 75%, compared with approximately 20% in control groups (Suntrup et al., 2015), and increased salivary SP levels were correlated with successful decannulation (Zhang et al., 2023). In addition, PES reduced nasogastric tube duration and length of hospital stay (Bath et al., 2016). Higher success rates were reported in younger patients, those receiving early intervention, those with supratentorial stroke, and those with better baseline feeding status (Cheng et al., 2024). A secondary analysis in ICU patients with tracheal intubation further showed that PES reduced the incidence of pneumonia and reintubation (Koestenberger et al., 2020). Modified mPES achieved a decannulation rate of approximately 50% in tracheostomized patients and significantly improved oral feeding safety, reduced PAS scores, and decreased pyriform sinus residue (Zhang et al., 2023). Experimental studies in brainstem ischemia models suggested that mPES might promote myelin and synaptic regeneration by regulating oligodendrocyte differentiation (Tian et al., 2025). However, some patients might fail to derive additional benefit, potentially due to suboptimal stimulation intensity or BDNF Val homozygosity (Jayasekeran et al., 2010; Bath et al., 2020).

#### Transcutaneous vagus nerve stimulation

Transcutaneous vagus nerve stimulation (tVNS) enhanced pharyngolaryngeal sensorimotor reflexes and promoted the release of norepinephrine (NE) and acetylcholine (ACh) by activating the nucleus tractus solitarius–locus coeruleus pathway, thereby relieving cortical inhibition and inducing neural reorganization (Wang et al., 2023). Concurrently, tVNS upregulated the gene expression of brain-derived neurotrophic factor (BDNF) and fibroblast growth factor (FGF) (Yuan et al., 2019). Stimulation targets included the auricular region (concha and external auditory canal) and the cervical region (along the anterior border of the sternocleidomastoid muscle at the carotid pulsation site). Reported parameters comprised a frequency of 25 Hz and a pulse width of 0.5 ms; tolerable intensities were approximately 1.83 ± 0.5 mA for the auricular site (Wang et al., 2023) and 2.5–3 mA for the cervical site (Yuan et al., 2019). Preclinical animal studies demonstrated that transcutaneous auricular vagus nerve stimulation (ta-VNS) promotes white matter repair and microenvironmental restoration by inhibiting inflammatory pathways, such as TLR4/NF-κB, while enhancing angiogenesis and vascular endothelial growth factor (VEGF) expression (Long et al., 2022). Clinically, an RCT demonstrated that the ta-VNS group showed significantly greater improvements in Mann Assessment of Swallowing Ability (MASA) scores, tongue strength, and soft palate function compared with controls (*p* < 0.05) (Tian et al., 2025), while a case report described a patient with medullary infarction who transitioned from nasogastric feeding to full oral intake after 6 weeks of treatment, accompanied by reduced pyriform sinus and vallecular residue and a decreased risk of aspiration (Wang et al., 2023).

#### Acupuncture

Acupuncture, grounded in meridian theory, exerted therapeutic effects by stimulating specific acupoints to regulate the flow of qi and blood and modulate visceral function (Zhang et al., 2015). As a branch of meridian theory, meridian sinew theory focused on the soft tissue–based motor system composed primarily of muscles, tendons, and ligaments (Cao et al., 2022). Acupuncture activated a neuromuscular–cutaneous network rich in sensory nerve endings, transmitting peripheral signals via afferent fibers to central structures such as the spinal cord and brain (Qin et al., 2022), thereby contributing to multidimensional improvement in PSD (Qiu et al., 2022). A multicenter RCT showed that, by day 14, the proportion of acute PSD patients achieving FOIS ≥6 was 78.0% after 2 weeks of true acupuncture, compared with 57.0% in the non-acupoint shallow-needling sham group, suggesting that acupoint stimulation may significantly improve swallowing function response in acute PSD (Liu et al., 2026). Acupuncture was also shown to promote swallowing recovery and improve quality of life, with sustained effects lasting up to 6 months (Yao et al., 2022), and earlier intervention was associated with greater therapeutic benefit (Li et al., 2025). A cohort study further reported that scalp acupuncture targeting the anterior oblique line of the vertex-temporal region (MS6), parietal line I (MS8), and parietal line II (MS9), combined with body acupuncture, yielded superior outcomes compared with rehabilitation alone (Mao et al., 2016). Acupuncture was frequently combined with other therapies to synergistically enhance neuromuscular recovery; multiple RCTs combining acupuncture with rehabilitation (Zheng et al., 2019) or balloon dilation (Song et al., 2020) demonstrated improvements in swallowing function, reduction of local muscle tension, and facilitation of tissue remodeling.

With regard to acupoint selection, Lianquan (CV23) and Fengchi (GB20) were considered core points (Wong et al., 2012). The deep layer of Lianquan corresponded anatomically to hypoglossal and glossopharyngeal nerve fibers in the tongue root region (Cui et al., 2020), and its stimulation activated swallowing-related muscles such as the genioglossus and thyrohyoid muscles (Zhang et al., 2024). Tongue acupuncture at Juquan and Haiquan points modulated cortical and thalamic activity, promoting collateral circulation and neural circuit reorganization (Cai et al., 2015). The “three sublingual needles” technique (CV23 and two points located 1 cun lateral to CV23 on both sides) was shown to decrease apparent diffusion coefficient (ADC) values and increase fractional anisotropy (FA) values in infarcted temporal regions (Zheng et al., 2023). Rapid needling at hyoid and laryngeal points (Li et al., 2017), as well as deep needling at Lianquan (Meng and Wang, 2015), contributed to improved epiglottic and laryngeal closure. Yamamoto New Scalp Acupuncture (YNSA) facilitated coordinated improvements across multiple functional domains by stimulating points corresponding to the hypoglossal and glossopharyngeal nerves (Zeise-Süss, 2011). Mechanistically, acupuncture exerted effects through both central and peripheral pathways: centrally, it promoted reorganization of key neural circuits, including M1 L5–PBN–NTS (Yao et al., 2023; Ye et al., 2024) and NTS–VPM–S1 pathways (Dai et al., 2025), activated paraventricular hypothalamic (PVH) neurons (Yuan et al., 2022), and modulated the hypoglossal nerve via M1 pyramidal neurons while enhancing SP release (Wong et al., 2012). Peripherally, acupuncture regulated local blood flow via transient receptor potential vanilloid 1 (TRPV1) channels (Yuan et al., 2024) and restored perineuronal nets (PNNs) in the peri-infarct M1 region (Yuan et al., 2023). Furthermore, electroacupuncture at Lianquan significantly increased c-Fos expression and BDNF levels in the contralateral M1, with 2 Hz identified as the optimal stimulation frequency (Yao et al., 2020). Fengchi (GB20), located adjacent to the vertebral artery and medullary swallowing center (Long et al., 2022), might enhance brainstem regulatory function by improving vertebrobasilar blood flow (Yang et al., 2024). Functional near-infrared spectroscopy (fNIRS) studies indicated that the tongue tri-needle technique might exert top-down modulation through the right dorsolateral prefrontal cortex (R_DLPFC) via integration of multiple brain networks (Sun et al., 2025). In addition, acupuncture at points such as Tiantu (CV22), Fengfu (GV16), Yifeng (SJ17), and Lianquan (CV23) improved swallowing function, nutritional indices (e.g., hemoglobin), and neurotransmitter levels (e.g., serotonin and dopamine) (Bai et al., 2024; Qin et al., 2025), while promoting neuroplasticity (Guo et al., 2018; Zhang et al., 2016). Acupoint injection (e.g., Xingnaojing at Fengchi) might further improve medullary function by modulating TXB2/PGF1α balance (Liu and Chen, 2017).

#### Peripheral sensory stimulation

Peripheral sensory stimulation included cold and thermal-tactile stimulation, as well as chemical stimulation (e.g., citric acid, capsaicin, and menthol), exerting its effects by enhancing central regulatory function within the cerebral cortex. Thermal tactile oral stimulation (TTOS) activated oropharyngeal sensory afferent pathways and induced short-term cortical plasticity. A magnetoencephalography study in healthy participants demonstrated that TTOS significantly enhanced bilateral cortical activation during swallowing—particularly in the left somatosensory cortex—and improved coordination between the oral and pharyngeal phases (Teismann et al., 2009). Ice stimulation enhanced the sensory sensitivity of the tongue and soft palate through combined thermal and mechanical inputs. An RCT showed that the ice stimulation group achieved significantly higher rates of swallowing recovery than the rehabilitation group, accompanied by reduced peripheral blood S100β levels, suggesting that it might promote recovery by attenuating central nervous system injury (Li et al., 2017). In addition, it might activate dormant neurons and promote neural network reconstruction, thereby facilitating functional reorganization. Basic studies further demonstrated that infusion of citric acid or capsaicin into the pharyngeal region significantly increased swallowing frequency in rat models of cerebral ischemia, indicating that peripheral chemosensory input represented a key pathway in the modulation of the swallowing reflex (Sugiyama et al., 2014). Collectively, these interventions enhanced peripheral sensory input to optimize central integration, constituting an important strategy for neuromodulation in PSD.

### Central neural regulation techniques

This section describes two intervention approaches that target the cerebral cortex and swallowing centers. By modulating cortical excitability, these approaches indirectly influence the neural networks underlying swallowing, thereby improving PSD.

#### Transcranial magnetic stimulation

Transcranial magnetic stimulation (TMS) modulated neuronal membrane potentials through pulsed magnetic fields (Sheng et al., 2023), mimicked endogenous patterns of neural discharge (Jiang et al., 2024) and generated induced electric fields that triggered axonal depolarization (Wang et al., 2021), thereby enabling precise regulation of cortical excitability. Repetitive TMS (rTMS) exerted its effects primarily on the basis of the hemispheric competition model. Low-frequency stimulation (≤1 Hz) suppressed cortical excitability (Cheng et al., 2021; Sun et al., 2023), and evidence suggested that low-frequency stimulation over the unaffected mylohyoid cortical representation could modulate cortical excitability in both hemispheres simultaneously (Du et al., 2016; Verin and Leroi, 2009). In contrast, high-frequency stimulation enhanced the excitability of the pharyngeal motor cortex in patients with acute PSD (Wu et al., 2024). For example, 3 Hz stimulation over the ipsilesional esophageal cortical representation improved oral intake and pharyngeal response time (Khedr et al., 2009), whereas bilateral 3 Hz stimulation (Wang et al., 2025) or 5 Hz stimulation (Zou et al., 2023) over the mylohyoid cortical representation increased bilateral MEP amplitudes. Other studies, based on the compensatory role of the unaffected hemisphere, further showed that 5 Hz rTMS over the unaffected hemisphere could regulate activity within the sensorimotor cortical network (Chen et al., 2025). Currently, no consensus has been reached on the optimal high-frequency stimulation protocol. Cerebellar rTMS (10 Hz, 250 pulses) significantly reversed suppression of pharyngeal motor evoked potentials (PMEPs) and swallowing accuracy induced by a virtual lesion, while enhancing corticobulbar excitability (Sasegbon et al., 2019; Vasant et al., 2015); a twice-daily protocol administered over 5 consecutive days appeared to yield superior outcomes (Wilkinson et al., 2025). Stimulation intensity was typically set at 80–120% of resting motor threshold (RMT) (Jefferson et al., 2009). However, the optimal high-frequency stimulation frequency remains to be definitively established. In terms of pulse number, 250 pulses represented the most commonly used and clinically effective rTMS setting (Sasegbon et al., 2019; Dong et al., 2022; Vasant et al., 2019), whereas intermittent theta burst stimulation (iTBS) mimicked hippocampal firing patterns and achieved more sustained increases in cortical excitability with 600 pulses (Rao et al., 2022; Han et al., 2025; Yu-Lei et al., 2022). No consensus has been reached on the optimal stimulation site: Cerebral rTMS targets included the cortical representations of the suprahyoid muscle group (e.g., the mylohyoid; Jiahui et al., 2023), as well as the pharyngeal (Jiao et al., 2022) and esophageal (Khedr et al., 2008)cortical representation areas, which were typically located 2–4 cm anterior and 3–10 cm lateral to Cz. Cerebellar targets were positioned 2.5 cm inferior and 4.3 cm lateral to the external occipital protuberance (Zhong et al., 2023). Cerebellar rTMS might regulate communication along the cerebellar-thalamic-cortical pathway and with the contralateral motor cortex (Dai et al., 2023), and might also influence both the cortex and the brainstem central pattern generator (CPG) through the dentate–tegmental pathway (Vasant et al., 2019; Sasegbon et al., 2020). Cerebellar iTBS was likewise been shown to enhance functional connectivity between the prefrontal and sensorimotor cortices (Zhang et al., 2025). An RCT demonstrated that 5 Hz rTMS applied to either the unaffected or affected hemisphere, as well as cerebellar rTMS, effectively improved swallowing function, with cerebellar stimulation also showing a favorable safety profile (Zhong et al., 2021). In addition, magnetic modulation targeting the left mastoid region to influence the vagus nerve was reported to improve cricopharyngeal MEP amplitude and latency after brainstem stroke, representing a novel therapeutic strategy (Lin et al., 2018). Paired associative stimulation (PAS), which combined peripheral nerve stimulation with stimulation of the corresponding muscle representation in the unaffected cortex, can effectively reverse bilateral cortical inhibition, improve swallowing behavior, and reduce aspiration (Michou et al., 2012).

Mechanistically, rTMS could modulate glutamate levels and attenuate excitotoxicity (Poh et al., 2019), thereby attenuating reperfusion injury and promoting salvage of the ischemic penumbra. It also upregulated substance P, calcitonin gene-related peptide (CGRP), and 5-hydroxytryptamine (5-HT), thereby enhancing the swallowing and cough reflexes (Chen et al., 2024), while promoting the release of neurotrophic factors such as BDNF. In terms of immunomodulation, rTMS might induce astrocytes and microglia to shift toward the protective A2 and M2 phenotypes (Sugiyama et al., 2014), and might additionally regulate intestinal epithelial function and microbial metabolism through the brain-gut axis (Zhao et al., 2024), thereby improving nutritional status, including serum albumin (ALB), hemoglobin (Hb), serum total protein (STP), and body mass index (BMI) (Jiao et al., 2023). Combining rTMS with TDT (Tarameshlu et al., 2019; Wen et al., 2023) or NMES (Zhang et al., 2019) might further enhance therapeutic efficacy. Notably, the therapeutic response to rTMS varied across individuals and appeared to be associated with COMT (rs6269) and DRD2 (rs1800497) polymorphisms (Raginis-Zborowska et al., 2019), as well as with the structural integrity of the corticobulbar tract (Im et al., 2020; Wang et al., 2023).

#### Transcranial direct current stimulation

Transcranial direct current stimulation (tDCS) activated large-scale cortical networks associated with swallowing via weak direct currents (Wang et al., 2023) and modulated transmembrane neuronal potentials (Zhao et al., 2022). This technique primarily operated via a compensatory mechanism, whereby anodal stimulation enhanced excitability of the unaffected hemisphere by depolarizing neuronal membranes, thereby compensating for functional deficits in the lesioned hemisphere (Cenci et al., 2024). Anodal stimulation was typically applied to key regions of the swallowing cortex, including the pharyngeal motor cortex in the contralesional hemisphere (midpoint of C3/T3 or C4/T4) (Suntrup-Krueger et al., 2018), the supramarginal gyrus of the unaffected hemisphere (Farpour et al., 2023), and the left mastoid region targeting the vagus nerve (Yan et al., 2025). In patients with cricopharyngeal dysfunction, bilateral esophageal cortical representations might also be selected (Wang et al., 2020). Given the right-hemisphere lateralization observed in brainstem stroke, some studies employed right-sided anodal stimulation (Suntrup-Krueger et al., 2018). Bilateral stimulation strategies were also explored to achieve greater increases in cortical excitability (Li et al., 2020; Duan et al., 2025). Cathodal stimulation was typically applied over the contralateral supraorbital region or the shoulder, reducing cortical excitability through hyperpolarization (Li et al., 2021). Treatment protocols commonly involved one session per day for 5–10 consecutive days, with cumulative effects associated with improved outcomes. One RCT demonstrated that 10 days of anodal stimulation over the lesioned hemisphere resulted in greater improvements in swallowing function in patients with chronic PSD (Shigematsu et al., 2013). Regarding stimulation parameters, the current intensity was typically set at 1–2 mA, and higher intensities (1.6–2 mA) showed superior efficacy (He et al., 2022). Other evidence has suggested that anodal stimulation at 1.4 mA for 20 min might be the optimal combined protocol (Xie et al., 2023). At the cellular and molecular levels, tDCS modulated sodium and calcium channel conductance, and its long-term effects were closely associated with N-methyl-D-aspartate (NMDA) receptor activity (Zhao et al., 2015). In addition, it induced long-term neurochemical changes in *γ*-aminobutyric acid (GABA) activity and modulated the synaptic microenvironment (Vasant et al., 2014). In combined therapeutic approaches, tDCS might establish functional coupling between network-level motor output and brain stimulation when integrated with conventional swallowing therapy (Mao et al., 2022), speech therapy (Farpour et al., 2023), and respiratory training (Li et al., 2023), thereby producing synergistic effects (Sawan et al., 2020). Such combined strategies demonstrated superior outcomes compared with monotherapy in swallowing function, infection-related indicators, and nutritional status, and might be particularly beneficial for specific subtypes such as swallowing apraxia (Yuan et al., 2017). One RCT further confirmed that a triple-combination regimen of conventional therapy, NMES, and tDCS significantly outperformed conventional therapy alone (Konecny and Elfmark, 2018).

#### Other adjunctive or alternative interventions

Botulinum toxin (BTX) inhibited acetylcholine release at the neuromuscular junction, inducing transient muscle relaxation and chemical denervation, and was primarily used to treat pharyngeal-phase dysphagia secondary to cricopharyngeal muscle dysfunction (Liu et al., 2023). To ensure injection accuracy and safety, multiple image-guided techniques were employed in clinical practice, including ultrasound guidance, which enabled real-time visualization of needle tip positioning and adjacent vascular and neural structures; when combined with balloon dilatation, it facilitated precise localization of stenotic segments and was commonly applied in salivary gland and cricopharyngeal muscle injections (Wei et al., 2019). Endoscopic guidance allowed direct targeting of key regions such as the posterolateral aspect, and early intervention was shown to improve dietary status and nutritional indices following acute lateral medullary infarction (Kang et al., 2019), whereas CT with esophageal balloon fluoroscopy enabled precise localization of injection sites, thereby minimizing the risk of inadvertent injury (Huai et al., 2020). Electromyography (EMG)-guided techniques were also employed; however, localization of the distal portion of the cricopharyngeal muscle might necessitate repeated EMG needle insertions, thereby increasing procedure-related risk (Alfonsi et al., 2017). An RCT demonstrated that BTX combined with rehabilitation therapy significantly improved dysphagia following posterior circulation stroke, with superior videofluoroscopic dysphagia scale (VDS) scores maintained at 3 months compared with rehabilitation alone (Chattopadhyay et al., 2023). BTX was also shown efficacy in acute ischemic PSD (Gusev et al., 2025), with therapeutic effects lasting for several months or longer, and case reports suggested that, in patients with persistent post-stroke cricopharyngeal dysfunction, a single BTX injection combined with rehabilitation might sustain swallowing improvement for up to 24 months, possibly due to the establishment of a positive functional feedback loop following reduction of UES pressure (Masiero et al., 2006). With regard to safety, caution was warranted due to potential diffusion of the toxin to adjacent inferior pharyngeal constrictor or laryngeal muscles, which might lead to adverse effects; observational studies suggested that patients who did not respond to the initial injection might be at increased risk of serious adverse events, such as aspiration pneumonia, with repeat injection, and that approximately 15% of patients might experience transient worsening of dysphagia or hoarseness (Huai et al., 2020).

## Discussion

### Key rehabilitation technique selection for different clinical stages of PSD

Rehabilitation strategies for PSD varied by swallowing stage. The oral phase was responsible for bolus formation and propulsion and involved the orbicularis oris, buccinator, masseter, palatal muscles, and intrinsic and extrinsic tongue muscles. The core intervention at this stage was oral motor training, which comprehensively engaged these muscle groups—for example, lip exercises targeted the orbicularis oris, while progressive tongue resistance training strengthened the tongue musculature. Neuromuscular electrical stimulation applied over the masseter and orbicularis oris region (Oh et al., 2017) could selectively improve oral-phase function, and acupuncture at points such as Lianquan (CV23), Jinjin (EX-HN12), and Yuye (EX-HN13) could directly modulate tongue and perioral muscle activity. The pharyngeal phase represented the central stage of the swallowing reflex and required coordinated laryngeal elevation, airway protection, and opening of the esophageal inlet. This phase involved four key muscle groups: the suprahyoid muscles (digastric, mylohyoid, and geniohyoid), which drove anterior–superior movement of the hyolaryngeal complex; the infrahyoid muscles, which contributed to laryngeal stabilization; the pharyngeal constrictor muscles, which generated peristaltic contraction; and the cricopharyngeal muscle, which regulated bolus passage into the esophagus. For the suprahyoid muscles, chin tuck against resistance (CTAR) was a primary intervention, while respiratory muscle training provided essential biomechanical support for swallowing by enhancing respiratory drive and intra-abdominal pressure, thereby facilitating suprahyoid muscle activation. Shaker exercise strengthened both supra- and infrahyoid muscles through isometric contraction, neuromuscular electrical stimulation could serve as an adjunct to facilitate activation, and the Mendelsohn maneuver improved hyolaryngeal excursion by voluntarily prolonging laryngeal elevation. For cricopharyngeal dysfunction, balloon dilatation provided direct mechanical expansion, whereas botulinum toxin injection alleviated muscle spasm; acupuncture at points such as Lianquan (CV23) and Fengchi (GB20) could regulate the timing and coordination of pharyngeal muscle contractions during the pharyngeal phase. During the esophageal phase, bolus transport depends on coordinated peristalsis of both striated and smooth esophageal muscles. The smooth muscle component is primarily regulated by the autonomic and enteric nervous systems (Goyal and Chaudhury, 2008), distinguishing it fundamentally from the oral and pharyngeal phases, which are under direct central neural control. Isolated esophageal-phase PSD is infrequently reported in the studies reviewed. Balloon dilation and botulinum toxin injection are commonly employed to relieve cricopharyngeal spasm or upper esophageal sphincter obstruction, facilitating bolus passage into the esophagus. For patients with prolonged nasogastric feeding who develop secondary gastroesophageal reflux, mucosal protection with proton pump inhibitors may be indicated (Liu et al., 2018). In cases of reduced esophageal body motility, current management strategies mainly involve dietary modifications and the use of prokinetic agents (Jandee et al., 2021).

**Figure 4:**
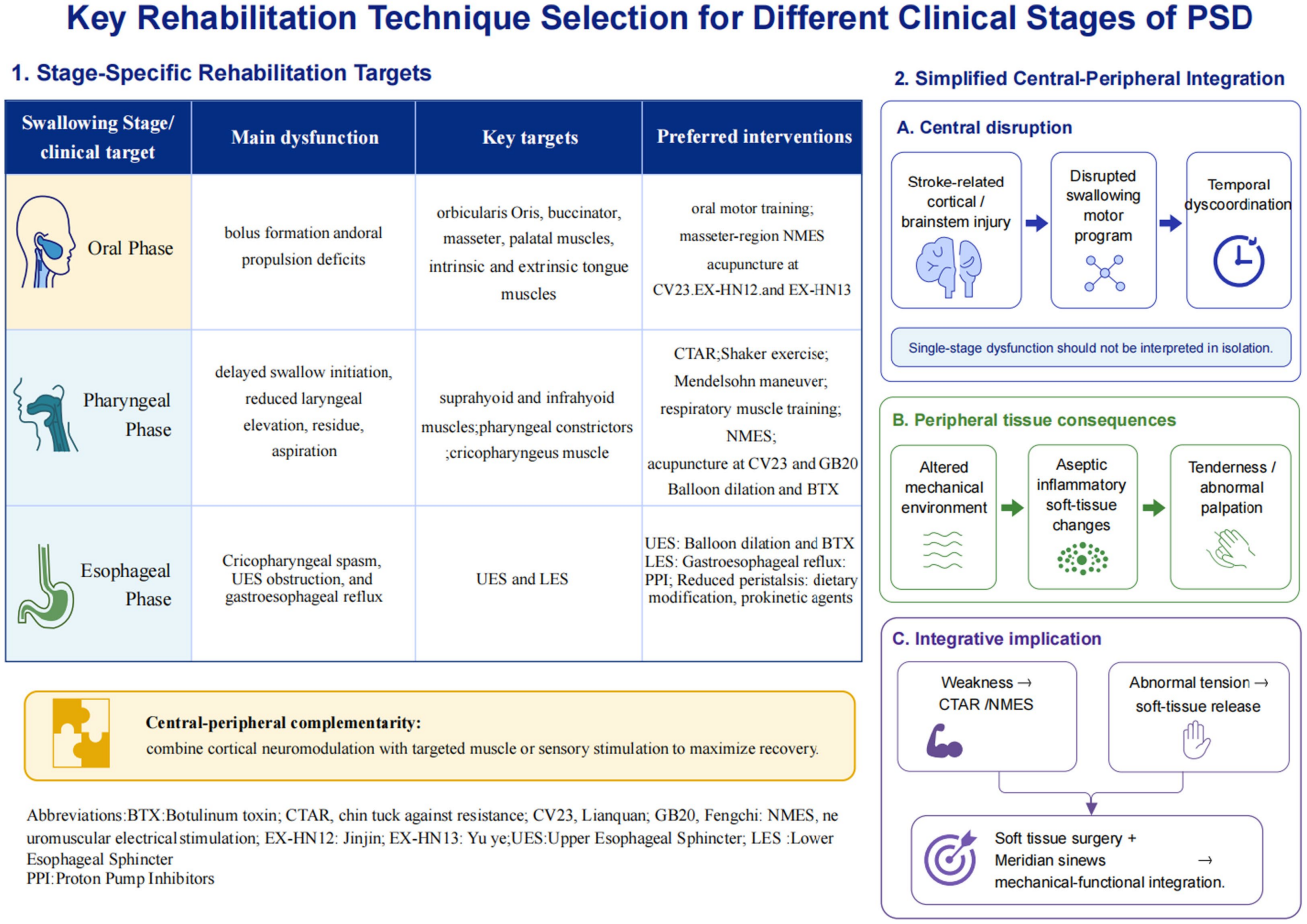
Key rehabilitation technique selection for different clinical stages of PSD.

When a stroke occurred, damage to the cerebral cortex or brainstem could disrupt or directly impair the swallowing central pattern generator (CPG), compromising this highly precise sequential motor program. Some muscle groups might become hypertonic and spastic, while others exhibited hypotonia and flaccidity, resulting in overall temporal dyscoordination. Therefore, dysfunction at any single swallowing stage should not be viewed in isolation. Soft tissue surgery emphasized the coordinated interaction among muscles and fascial soft tissues (Lu et al., 2024; Hu et al., 2021). Post-stroke alterations in the mechanical environment could induce aseptic inflammatory changes in local soft tissues of the tongue, palate, and pharynx (Coelho Junior et al., 2016), potentially leading to inflammatory cell infiltration in adipose tissue and thickening of the muscle layer (Xuan, 2009; Tidball, 2011), with clinical manifestations of localized tenderness or abnormal palpation findings. For instance, when weakness of the suprahyoid muscle group prevented anterior–superior elevation of the hyolaryngeal complex, exercise-based interventions such as CTAR could be applied to strengthen the hypotonic muscles, or NMES could be used to enhance peripheral neural drive. Concurrently, palpation could identify abnormal tension in antagonistic or synergistic muscles, which could then be released to restore mechanical balance. This perspective aligned with the holistic view of acupuncture’s 12 Meridian Sinews, which organized the body’s musculotendinous network into 12 functional pathways. However, most acupuncture RCTs cited in this review did not assess blinding adequacy, and sham-controlled acupuncture trials carried an acknowledged high risk of bias due to insufficient blinding. If soft tissue surgery and meridian sinew theory could provide strictly reproducible operational standards for acupoint selection and stimulation methods, they might offer novel insights for the clinical optimization and integration of neuromuscular stimulation techniques (Figure 4).

#### Complementary strategies for central and peripheral neuromodulation

Rehabilitation of PSD required precise neuromodulation spanning central and peripheral levels. Central regulation involved brainstem-mediated neural output to the swallowing muscles, initiating voluntary swallowing and regulating the oral and pharyngeal phases, after which swallowing activity was primarily governed by CPG neurons within the brainstem (Sasegbon et al., 2025). Key techniques included repetitive transcranial magnetic stimulation (rTMS) and transcranial direct current stimulation (tDCS), both non-invasive brain stimulation (NIBS) techniques. rTMS utilized magnetic fields to generate induced currents that directly triggered neuronal discharge, whereas tDCS modulated resting membrane potential via direct current without directly inducing neuronal depolarization (Shibata et al., 2023). tDCS electrodes covered a broader cortical area but had a shallower penetration depth (approximately 1 cm), whereas figure-of-eight coil rTMS achieved depths exceeding 2 cm, with H-coils reaching up to 5.5 cm (Qiao et al., 2025). TMS allowed millisecond-scale single-pulse stimulation, whereas tDCS did not (Pisegna et al., 2016). TMS was primarily based on the interhemispheric inhibition model, restoring balance by either suppressing excessive excitation in the unaffected hemisphere at low frequencies or activating the affected hemisphere at high frequencies, whereas tDCS was based on a compensatory model that enhanced the function of unaffected regions (Oh et al., 2017). Regarding safety, rTMS carried an extremely low seizure incidence (<0.1%) and was considered safe for patients with pacemakers (Cheng and Hamdy, 2021), although it involved costly equipment and higher technical demands (Labeit et al., 2024), whereas tDCS was suitable for bedside application and could be delivered concurrently with rehabilitation training (Yang et al., 2012). Peripheral stimulation enhanced sensory input to activate and optimize the brainstem swallowing center: pharyngeal electrical stimulation activated glossopharyngeal and vagus afferent fibers by stimulating the pharyngeal mucosa, inducing substance P release and delivering potent sensory input to the nucleus tractus solitarius, thereby reshaping swallowing reflex excitability; vagus nerve stimulation activated the nucleus tractus solitarius–locus coeruleus pathway via auricular or cervical targets, promoting norepinephrine and acetylcholine release; acupuncture directly targeted regions rich in glossopharyngeal, vagus, and hypoglossal nerve endings; and peripheral sensory stimulation enhanced oropharyngeal sensory feedback via trigeminal and glossopharyngeal afferent activation.

**Figure 5:**
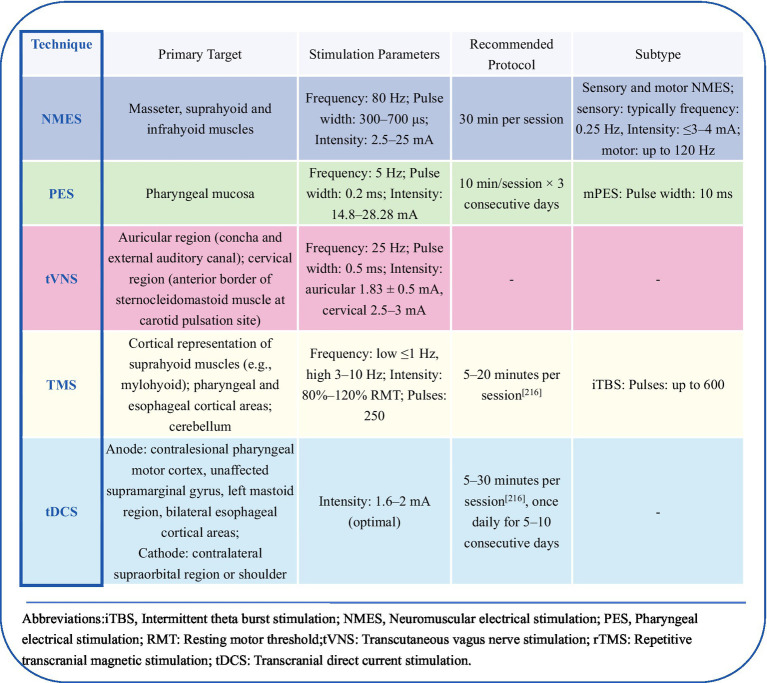
Stimulation parameters for peripheral and central interventions.

**Figure 6:**
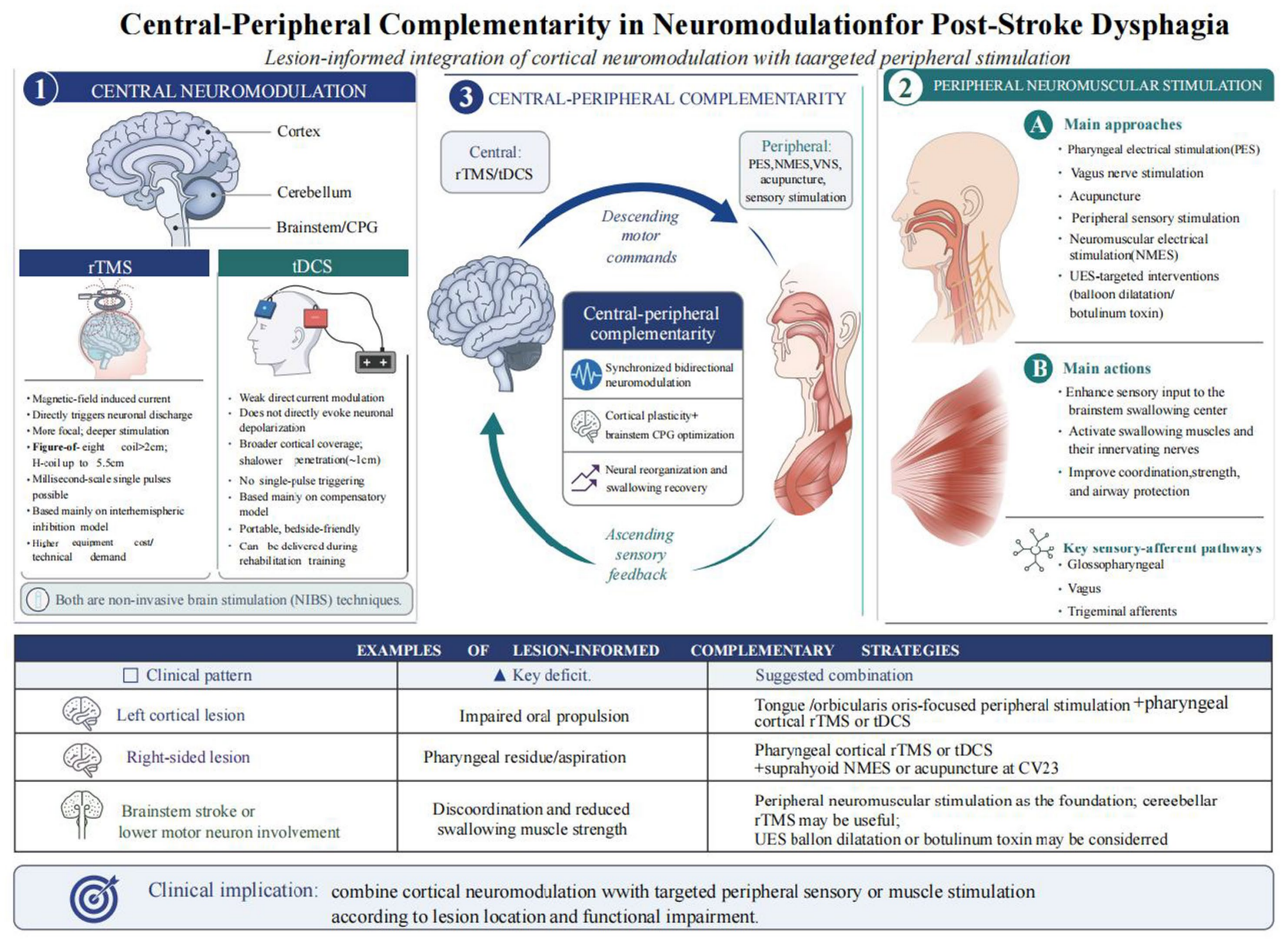
Central-peripheral complementarity in neuromodulation for PSD.

Due to differences in the targets of central and peripheral neuromodulation, peripheral neuromuscular stimulation primarily acted on agonist muscles and their innervating nerves, whereas central neuromodulation primarily modulated cortical swallowing representation areas. However, most of the included studies combined these approaches with conventional swallowing rehabilitation therapy, and clinical trials integrating central and peripheral neuromodulation remained scarce. Therefore, this study proposed that neuromuscular-related interventions for PSD might be applied as complementary strategies in combination. Upper motor neurons predominantly regulated swallowing function. For example, anatomical and pathophysiological analyses indicated that the left cerebral cortex was more involved in oral-phase control, whereas right-sided lesions were more closely associated with pharyngeal residue and aspiration (Suntrup and Dziewas, 2013; Robbins et al., 1993). Meanwhile, targeted clinical strategies could be formulated by integrating imaging findings with functional assessments: for left-sided lesions accompanied by impaired oral propulsion, peripheral interventions targeting the tongue muscles and orbicularis oris could be strengthened alongside pharyngeal cortical stimulation; for prolonged pharyngeal phase or aspiration, pharyngeal cortical rTMS or tDCS combined with suprahyoid muscle NMES or acupuncture at Lianquan (CV23) might form a complementary therapeutic approach. Damage to lower motor neurons impaired motor output to swallowing muscles, requiring rTMS to exert indirect effects via residual neural networks (Cheng et al., 2025); in such cases, cerebellar rTMS demonstrated advantages by modulating the cerebellum–thalamus–cortex swallowing network (Zhong et al., 2023). Brainstem stroke was characterized by discoordination across swallowing phases and reduced muscle strength; therefore, peripheral neuromuscular stimulation served as a fundamental approach to improve coordination and enhance muscle strength. For example, four-channel NMES could improve coordination of the hyolaryngeal complex, while balloon dilatation of the UES or botulinum toxin injection could relieve cricopharyngeal muscle spasm. The essence of central–peripheral complementarity lay in bidirectional neural modulation: when descending motor commands and sensory feedback were highly synchronized in both time and space, they most effectively induced specific reorganization and strengthening of spinal and cortical neural circuits, thereby promoting recovery of swallowing function (Figures 5, 6).

#### Future directions for research

Future research should extend beyond single-technique validation to embrace integrated approaches that combine central neuromodulation, peripheral nerve stimulation, and training of the muscles they innervate. Intervention strategies should be tailored along neuromuscular pathways based on lesion location, disease stage, and dysphagia subtype. This implies that, although conventional randomized controlled trials (RCTs) remain the cornerstone of evidence generation, complementary research methodologies are also needed. Traditional RCTs rely on random allocation to minimize bias and determine intervention efficacy; however, PSD is highly heterogeneous, with substantial interindividual differences in lesion characteristics and clinical presentation. Therefore, alongside large-scale RCTs that provide high-level evidence, in-depth case series, single-case experimental designs, and studies based on real-world data will serve as valuable complementary approaches. Achieving dynamic combinatorial interventions will require technological advances, including the integration of multimodal neuroimaging, neurophysiological assessments, and synchronized biomechanical measurements to enable dynamic monitoring of neural pathway changes before and after intervention. At the same time, establishing a real-time closed-loop framework of “assessment–intervention–reassessment” will facilitate the transition of rehabilitation paradigms from static protocols to adaptive, responsive strategies. Ultimately, the goal is to elucidate the mechanistic framework underlying central–peripheral interactions and to develop precision interventions capable of real-time sensing of neuromuscular states and adaptive adjustment accordingly.

## Conclusion

While several systematic reviews and meta-analyses have examined individual interventions for PSD, the present review provides a comprehensive, integrated framework that incorporates neuromuscular mechanisms, soft-tissue pathology, and individualized treatment pathways across different clinical stages. Based on the present systematic analysis, the future of PSD management lies in shifting clinical practice from generalized protocols toward deeply integrated, individualized pathways. This requires rehabilitation strategies to be strictly guided by a precise characterization of dysphagia stage and the nature of neural injury, thereby enabling the rational integration of targeted central neuromodulation and peripheral interventions. In addition, incorporating a neuromuscular soft–tissue coordination perspective calls for an in-depth investigation of the full pathological continuum—from central command dysregulation to secondary peripheral muscle inflammation and biomechanical imbalance—as well as the interactions among these processes. Achieving this objective depends on the parallel development of objective, fine-grained quantitative assessment techniques and intelligent intervention systems capable of dynamic, feedback-driven adaptation. Ultimately, through rigorous integration of precise evaluation, mechanistic insights, and adaptive interventions—supported by in-depth case series and high-quality evidence synthesis—a comprehensive, clinically applicable, personalized treatment framework for PSD can be established.

## Data Availability

The original contributions presented in the study are included in the article/Supplementary material, further inquiries can be directed to the corresponding authors.
