# Supplementary material for: Neuromuscular-related interventions for post-stroke dysphagia: a comprehensive narrative review
Source: Front Neurosci. 2026 Jun 24;20:1824363. doi: 10.3389/fnins.2026.1824363 (PMC13341806; doi:10.3389/fnins.2026.1824363)
Supplement: Supplementary file 1 [file Table_1.DOCX]

Supplementary materials 1 Retrieval

Pubmed

| No. | Search summary |
| --- | --- |
| #1 | (Oral exercise[Title/Abstract] OR oral motor exercise[Title/Abstract] OR oral training[Title/Abstract]) |
| #2 | (chin tuck against resistance[Title/Abstract] OR CTAR[Title/Abstract] OR chin tuck exercise[Title/Abstract]) |
| #3 | (Chin-down posture[Title/Abstract] OR chin tuck posture[Title/Abstract] OR chin-down maneuver[Title/Abstract] OR chin tuck maneuver[Title/Abstract]) |
| #4 | (Mendelsohn Maneuver[Title/Abstract]) |
| #5 | (Masako Manoeuvre[Title/Abstract]) |
| #6 | (Shaker exercise[Title/Abstract] OR Shaker training[Title/Abstract]) |
| #7 | (Breathing Exercises[MeSH Terms]) OR (respiratory muscle training[Title/Abstract] OR inspiratory muscle training[Title/Abstract] OR expiratory muscle training[Title/Abstract]) |
| #8 | (Biofeedback, Psychology[MeSH Terms]) OR (EMG biofeedback[Title/Abstract] OR electromyographic biofeedback[Title/Abstract] OR surface electromyographic biofeedback[Title/Abstract] OR sEMG biofeedback[Title/Abstract]) |
| #9 | (Transcutaneous Electric Nerve Stimulation[MeSH Terms]) OR (TENS[Title/Abstract] OR transcutaneous electrical nerve stimulation[Title/Abstract] OR percutaneous electrical nerve stimulation[Title/Abstract] OR neuromuscular electrical stimulation[Title/Abstract]) |
| #10 | (pharyngeal electrical stimulation[Title/Abstract] OR Modified pharyngeal electrical stimulation[Title/Abstract]) |
| #11 | (Vagus Nerve Stimulation[MeSH Terms]) OR (vagal nerve stimulation[Title/Abstract] OR VNS[Title/Abstract]) |
| #12 | (Acupuncture[MeSH Terms] OR Electroacupuncture[MeSH Terms]) |
| #13 | (Capsaicin[MeSH Terms]) OR (Capsaicin[Title/Abstract]) |
| #14 | (Menthol[MeSH Terms]) OR (Menthol[Title/Abstract]) |
| #15 | (Piperine[Title/Abstract] OR 1-piperoylpiperidine[Title/Abstract]) |
| #16 | (Transcranial Magnetic Stimulation[MeSH Terms]) OR (TMS[Title/Abstract] OR repetitive transcranial magnetic stimulation[Title/Abstract]) |
| #17 | (Transcranial Direct Current Stimulation[MeSH Terms]) OR (tDCS[Title/Abstract] OR transcranial direct current stimulation[Title/Abstract]) |
| #18 | (Botulinum Toxins[MeSH Terms]) OR (Botulinum toxin[Title/Abstract] OR Botox[Title/Abstract]) |
| #19 | #1 AND #2 AND #3 AND #4 AND #5 AND #6 AND #7 AND #8 AND #9 AND #10 AND #11 AND #12 AND #13 AND #14 AND #15 AND #16 AND #17 AND #18 |
| #20 | (Stroke[MeSH Terms]) OR (Stroke[Title/Abstract] OR Cerebrovascular Accident[Title/Abstract] OR Apoplexy[Title/Abstract] OR CVA[Title/Abstract]) |
| #21 | (Deglutition Disorders[MeSH Terms]) OR (Deglutition Disorders[Title/Abstract]) OR (Dysphagia[Title/Abstract] OR Swallowing disorder[Title/Abstract]) |
| #22 | #19 AND #20 AND #21 |

Embase

| No. | Search summary |
| --- | --- |
| #22 | #19 AND #20 AND #21 |
| #21 | 'cerebrovascular accident'/exp OR 'stroke':ti,ab,kw OR 'cerebrovascular accident':ti,ab,kw OR 'apoplexy':ti,ab,kw OR 'cva':ti,ab,kw |
| #20 | 'dysphagia'/exp OR 'dysphagia':ti,ab,kw OR 'swallowing disorder':ti,ab,kw |
| #19 | #1 AND #2 AND #3 AND #4 AND #5 AND #6 AND #7 AND #8 AND #9 AND #10 AND #11 AND #12 AND #13 AND #14 AND #15 AND #16 AND #17 AND #18 |
| #18 | 'botulinum toxin'/exp OR 'botulinum toxin':ti,ab,kw OR 'botox':ti,ab,kw |
| #17 | 'transcranial direct current stimulation'/exp OR 'tdcs':ti,ab,kw OR 'transcranial direct current stimulation':ti,ab,kw |
| #16 | 'transcranial magnetic stimulation'/exp OR 'tms':ti,ab,kw OR 'repetitive transcranial magnetic stimulation':ti,ab,kw |
| #15 | 'menthol'/exp OR 'menthol':ti,ab,kw |
| #14 | 'capsaicin'/exp OR 'capsaicin':ti,ab,kw |
| #13 | 'piperine'/exp OR 'piperine':ti,ab,kw OR '1-piperoylpiperidine':ti,ab,kw |
| #12 | 'acupuncture'/exp OR 'electroacupuncture'/exp |
| #11 | 'vagus nerve stimulation'/exp OR 'vagal nerve stimulation':ti,ab,kw OR 'vns':ti,ab,kw |
| #10 | 'pharyngeal electrical stimulation':ti,ab,kw OR 'modified pharyngeal electrical stimulation':ti,ab,kw |
| #9 | 'transcutaneous electrical nerve stimulation'/exp OR 'tens':ti,ab,kw OR 'transcutaneous electrical nerve stimulation':ti,ab,kw OR 'percutaneous electrical nerve stimulation':ti,ab,kw OR 'neuromuscular electrical stimulation':ti,ab,kw |
| #8 | 'biofeedback'/exp OR 'emg biofeedback':ti,ab,kw OR 'electromyographic biofeedback':ti,ab,kw OR 'surface electromyographic biofeedback':ti,ab,kw OR 'semg biofeedback':ti,ab,kw |
| #7 | 'breathing exercise'/exp OR 'respiratory muscle training':ti,ab,kw OR 'inspiratory muscle training':ti,ab,kw OR 'expiratory muscle training':ti,ab,kw |
| #6 | 'shaker exercise':ti,ab,kw OR 'shaker training':ti,ab,kw |
| #5 | 'masako manoeuvre':ti,ab,kw |
| #4 | 'mendelsohn maneuver':ti,ab,kw |
| #3 | 'chin-down posture':ti,ab,kw OR 'chin tuck posture':ti,ab,kw OR 'chin-down maneuver':ti,ab,kw OR 'chin tuck maneuver':ti,ab,kw |
| #2 | 'chin tuck against resistance':ti,ab,kw OR 'ctar':ti,ab,kw OR 'chin tuck exercise':ti,ab,kw |
| #1 | 'oral exercise':ti,ab,kw OR 'oral motor exercise':ti,ab,kw OR 'oral training':ti,ab,kw |

MEDILINE

| No. | Search summary |
| --- | --- |
| S22 | S19 AND S20 AND S21 |
| S21 | MM (Stroke) OR MH (Stroke) OR AB (Stroke OR Cerebrovascular Accident OR Apoplexy OR CVA) OR TI (Stroke OR Cerebrovascular Accident OR Apoplexy OR CVA) |
| S20 | MM (Deglutition Disorders) OR MH (Deglutition Disorders) OR AB (Dysphagia OR Swallowing disorder) OR TI (Dysphagia OR Swallowing disorder) |
| S19 | S1 AND S2 AND S3 AND S4 AND S5 AND S6 AND S7 AND S8 AND S9 AND S10 AND S11 AND S12 AND S13 AND S14 AND S15 AND S16 AND S17 AND S18 |
| S18 | MM(Botulinum Toxins) OR MH(Botulinum Toxins) OR AB(Botulinum toxin OR Botox) OR TI(Botulinum toxin OR Botox) |
| S17 | MM(Transcranial Direct Current Stimulation) OR MH(Transcranial Direct Current Stimulation) OR AB(tDCS OR transcranial direct current stimulation) OR TI(tDCS OR transcranial direct current stimulation) |
| S16 | MM(Transcranial Magnetic Stimulation) OR MH(Transcranial Magnetic Stimulation) OR AB(TMS OR repetitive transcranial magnetic stimulation) OR TI(TMS OR repetitive transcranial magnetic stimulation) |
| S15 | MM(Menthol) OR MH(Menthol) OR AB(Menthol) OR TI(Menthol) |
| S14 | MM(Capsaicin) OR MH(Capsaicin) OR AB(Capsaicin) OR TI(Capsaicin) |
| S13 | MM (Piperine) OR MH (Piperine) OR AB (Piperine OR 1-piperoylpiperidine) OR TI (Piperine OR 1-piperoylpiperidine) |
| S12 | MM (Acupuncture OR Electroacupuncture) OR MH (Acupuncture OR Electroacupuncture) OR AB (Acupuncture OR Electroacupuncture) OR TI (Acupuncture OR Electroacupuncture) |
| S11 | MM (Vagus Nerve Stimulation) OR MH (Vagus Nerve Stimulation) OR AB (vagal nerve stimulation OR VNS) OR TI (vagal nerve stimulation OR VNS) |
| S10 | MM (pharyngeal electrical stimulation) OR MH (pharyngeal electrical stimulation) OR AB (pharyngeal electrical stimulation OR modified pharyngeal electrical stimulation) OR TI (pharyngeal electrical stimulation OR modified pharyngeal electrical stimulation) |
| S9 | MM (Transcutaneous Electric Nerve Stimulation) OR MH (Transcutaneous Electric Nerve Stimulation) OR AB (TENS OR transcutaneous electrical nerve stimulation OR percutaneous electrical nerve stimulation OR neuromuscular electrical stimulation) OR TI (TENS OR transcutaneous electrical nerve stimulation OR percutaneous electrical nerve stimulation OR neuromuscular electrical stimulation) |
| S8 | MM (Biofeedback, Psychology) OR MH (Biofeedback, Psychology) OR AB (EMG biofeedback OR electromyographic biofeedback OR surface electromyographic biofeedback OR sEMG biofeedback) OR TI (EMG biofeedback OR electromyographic biofeedback OR surface electromyographic biofeedback OR sEMG biofeedback) |
| S7 | MM (Breathing Exercises) OR MH (Breathing Exercises) OR AB (respiratory muscle training OR inspiratory muscle training OR expiratory muscle training) OR TI (respiratory muscle training OR inspiratory muscle training OR expiratory muscle training) |
| S6 | MM (Shaker) OR MH (Shaker) OR AB (Shaker exercise OR Shaker training) OR TI (Shaker exercise OR Shaker training) |
| S5 | MM (Masako Manoeuvre) OR MH (Masako Manoeuvre) OR AB (Masako Manoeuvre) OR TI (Masako Manoeuvre) |
| S4 | MM (Mendelsohn Maneuver) OR MH (Mendelsohn Maneuver) OR AB (Mendelsohn Maneuver) OR TI (Mendelsohn Maneuver) |
| S3 | MM (Chin-down posture) OR MH (Chin-down posture) OR AB (Chin-down posture OR chin tuck posture OR chin-down maneuver OR chin tuck maneuver) OR TI (Chin-down posture OR chin tuck posture OR chin-down maneuver OR chin tuck maneuver) |
| S2 | MM (chin tuck against resistance) OR MH (chin tuck against resistance) OR AB (chin tuck against resistance OR CTAR OR chin tuck exercise) OR TI (chin tuck against resistance OR CTAR OR chin tuck exercise) |
| S1 | MM (Oral exercise) OR MH (Oral exercise) OR AB (Oral exercise OR oral motor exercise OR oral training) OR TI (Oral exercise OR oral motor exercise OR oral training) |

Web Of Science

Search summary **:**Oral exercise (Topic) or oral motor exercise (Topic) or oral training (Topic) or chin tuck against resistance (Topic) or CTAR (Topic) or chin tuck exercise (Topic) or Chin-down posture (Topic) or chin tuck posture (Topic) or chin-down maneuver (Topic) or chin tuck maneuver (Topic) or Mendelsohn Maneuver (Topic) or Masako Manoeuvre (Topic) or Shaker exercise (Topic) or Shaker training (Topic) or Breathing Exercises (Topic) or respiratory muscle training (Topic) or inspiratory muscle training (Topic) or expiratory muscle training (Topic) or EMG biofeedback (Topic) or electromyographic biofeedback (Topic) or surface electromyographic biofeedback (Topic) or sEMG biofeedback (Topic) or TENS (Topic) or transcutaneous electrical nerve stimulation (Topic) or percutaneous electrical nerve stimulation (Topic) or neuromuscular electrical stimulation (Topic) or pharyngeal electrical stimulation (Topic) or modified pharyngeal electrical stimulation (Topic) or Vagus Nerve Stimulation (Topic) or vagal nerve stimulation (Topic) or VNS (Topic) or Acupuncture (Topic) or Electroacupuncture (Topic) or Piperine (Topic) or 1-piperoylpiperidine (Topic) or Capsaicin (Topic) or Menthol (Topic) or Transcranial Magnetic Stimulation (Topic) or TMS (Topic) or repetitive transcranial magnetic stimulation (Topic) or Transcranial Direct Current Stimulation (Topic) or tDCS (Topic) or Botulinum toxin (Topic) or Botox (Topic) and Dysphagia (Topic) or Swallowing disorder (Topic) and Stroke (Topic) or Cerebrovascular Accident (Topic) or Apoplexy (Topic) or CVA (Topic)
